# Association of exposure to per- and polyfluoroalkyl substances with liver injury in American adults

**DOI:** 10.7555/JBR.38.20240018

**Published:** 2024-05-29

**Authors:** Yuqian Yan, Lu Zhang, Xin Xu, Jing Lu, Xinyuan Ge, Maojie Liu, Juan Yang, Chan Tian, Zijun Ge, Chengxiao Yu, Wen Guo, Chunyan Ye, Qun Zhang

**Affiliations:** 1 Health Management Center, the First Affiliated Hospital of Nanjing Medical University, Nanjing, Jiangsu 210029, China; 2 Department of Epidemiology, China International Cooperation Center on Environment and Human Health, Center for Global Health, School of Public Health, Nanjing Medical University, Nanjing, Jiangsu 211166, China; 3 Office of Infection Management, the First Affiliated Hospital of Nanjing Medical University, Nanjing, Jiangsu 210029, China; 4 Department of Liver Diseases, the Third People's Hospital of Changzhou, Changzhou, Jiangsu 213000, China

**Keywords:** PFASs, NHANES, liver function, WQS, liver injury

## Abstract

Epidemiological data are scarce regarding the association of exposure to mixtures of per- and polyfluoroalkyl substances (PFASs) with liver injury in the general population. In the current study, therefore, we examined data from the National Health and Nutrition Examination Survey (2009–2018). The PFAS exposure levels were defined by the serum concentrations of PFASs with over 70% detection in samples, namely perfluorooctanoic acid, perfluorononanoic acid, perfluorohexane sulfonic acid, perfluorodecanoic acid, and perfluorooctane sulfonic acid (PFOS). We evaluated liver injury from two aspects: first, the degree of liver inflammation was determined based on levels of the serum alanine aminotransferase, aspartate aminotransferase, glutamyltransferase, and total bilirubin; second, the degree of liver fibrosis was determined based on the fibrosis-4 index. We assessed the associations of individual or total PFAS exposure with these liver injury outcomes using multivariable linear and logistic regression models, restricted cubic splines, and weighted quantile sum regression. Among the samples of 7484 American adults, the median concentration of PFOS was the highest, followed by perfluorooctanoic acid and perfluorohexane sulfonic acid. Using multivariable linear regression, we observed positive correlations between all PFAS exposure and liver enzyme levels, such as alanine aminotransferase, aspartate aminotransferase, and total bilirubin. Additionally, the weighted quantile sum model indicated an overall positive association between exposure to the five PFASs and liver injury risk. For liver function biomarkers and liver fibrosis, perfluorononanoic acid and PFOS were the most heavily weighted chemicals, respectively. Our findings provide new epidemiological evidence indicating potential associations between PFAS exposure and adverse effects on liver injury biomarkers, highlighting the potentially harmful effects of PFAS exposure on human liver health.

## Introduction

Liver disease is a significant global health issue, affecting over 800 million individuals, and is a primary contributor to illness and death ^[ 1] ^. The liver, essential for metabolic balance, plays a crucial role in metabolizing, distributing, and removing external substances. Increasing evidence indicates that various environmental contaminants and chemicals are involved in causing liver damage and increasing the likelihood of compromised liver function ^[ 2] ^.

Per- and polyfluoroalkyl substances (PFASs), a broad range of man-made chemicals known for their alkyl chains, have been widely used in numerous commercial and industrial settings ^[ 3] ^. These chemicals possess unique physiochemical properties, such as high stability and persistence, as well as resistance to water, oil, stains, and grease, even in extreme temperatures and harsh environments ^[ 4] ^. Individuals may encounter PFASs through multiple means, including direct contact with non-stick cookware, food packaging, furnishings, firefighting foams, surfactants, lubricants, paints, polishes, and coatings on textiles ^[ 3] ^. Additionally, PFASs may be absorbed through the inhalation and ingestion of indoor air and household dust, and environmental elements like surface water, soil, and sediment also serve as exposure sources ^[ 5] ^. PFASs accumulate in various human organs, including the lungs, kidneys, brain, liver, bones, and placenta. Furthermore, these compounds have an extended biological half-life estimated to last as long as two to four years, allowing them to persist and threaten the organs ^[ 6] ^.

Previous studies consistently indicated that PFASs were hepatotoxic, as evidenced by their association with liver function markers ^[ 7– 8] ^. Furthermore, experiments on rodents indicated a potential causal association between PFASs and liver toxicity ^[ 9] ^. Based on existing limited *in vitro* and *in vivo* studies, there may be individual differences in the degree of PFAS compound effects on liver function, but the study results on the relative potency of these compounds were inconsistent ^[ 10– 11] ^. In addition, our understanding of the combined effects of PFASs on liver injury in adults remains scant, with limited assessment dimensions. Unlike exposure to a single type of PFAS, people encounter a mixture of these substances in everyday life. Individual PFAS exposure may not fully account for the development of liver injury, while neglecting multi-PFAS effects may cause biased estimates of the effects of a single PFAS ^[ 12– 13] ^. Therefore, exploring how different PFASs, both individually and as a mixture, are associated with liver injury in adults is important.

The current study focused on assessing both the individual and combined effects of five serum PFAS concentrations on liver injury in human adults, using data from the National Health and Nutrition Examination Survey (NHANES) conducted between 2009 and 2018.

## Subjects and methods

### Study design and population

The data used in the current study were sourced from a compilation of five NHANES cycles conducted between 2009 and 2018, including the 2009–2010, 2011–2012, 2013–2014, 2015–2016, and 2017–2018 cycles. NHANES, conducted by the National Center for Health Statistics (NCHS), primarily evaluates the health and nutritional status of the U.S. adult and child populations. The NCHS used stratified, clustered, multi-stage probability surveys to gather representative data from the non-institutionalized civilian populations in the U.S. ^[ 14] ^. The NHANES dataset is publicly available and freely downloadable from the website listed below: https://wwwn.cdc.gov/nchs/nhanes/Default.aspx. The current study was approved by the Institutional Review Board of the NCHS, and each participant provided a written consent.

**Figure 1:**
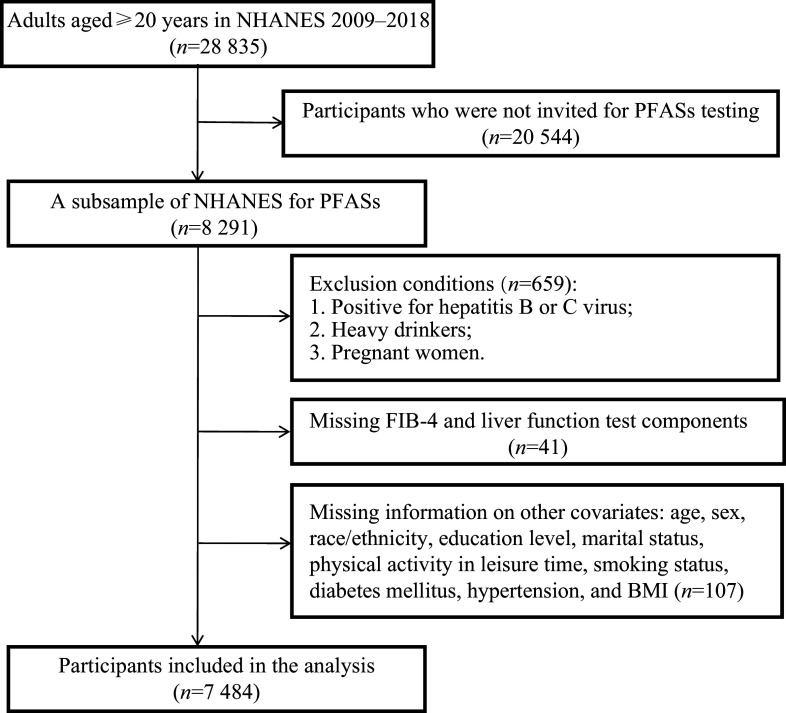
Flow chart of the selection process of the participants in the current study. Abbreviations: PFASs, per- and polyfluoroalkyl substances.

The current study included 28835 adults aged ≥ 20 years, enrolled based on age restrictions. A random subsample of 8291 participants had their PFAS serum concentrations measured. Participants with other liver disease causes, such as hepatitis B (confirmed by surface antigen test) or C (detected viral RNA and/or positive antibody test), and those with excessive alcohol consumption (> 30 g/day for men and > 20 g/day for women) were excluded. We also omitted individuals with incomplete covariate and outcome data, as well as pregnant participants. The final study population comprised 7484 adults. The flow chart of the selection process is shown in ***Fig. 1***.

### Outcome ascertainment

The evaluation of liver injury typically involves assessing liver inflammation through serum liver function tests and determining the degree of liver fibrosis ^[ 14] ^. Key biomarkers for liver function include alanine aminotransferase (ALT), aspartate aminotransferase (AST), gamma-glutamyltransferase (GGT), and total bilirubin (TBIL). The elevated levels of ALT or AST indicate damage or disease states in hepatocytes and myocardial cells ^[ 15] ^. GGT is an indicator of cholestasis, with elevated levels indicating liver damage, fatty liver, or oxidative stress ^[ 16] ^. TBIL, derived from heme breakdown, is measured in the serum, reflecting the balance between its production and hepatic clearance ^[ 17] ^. The fibrosis-4 (FIB-4) index, which serves as a non-invasive diagnostic tool for assessing liver fibrosis levels, is calculated using the following formula: (age × AST)/(platelets × ALT ^1/2^), where a value below 1.3 indicates low risk and a value of 1.3 or above indicates moderate-to-high risk of advanced liver fibrosis ^[ 18] ^.

For the period 2009–2016, abnormal levels of ALT (> 47 IU/L for men, > 30 IU/L for women), AST (> 33 IU/L for both sexes), GGT (> 65 IU/L for men, > 36 IU/L for women), and TBIL (> 1.3 mg/dL for both sexes) were defined using NHANES 2009–2016 upper reference limits. In 2017–2018, NHANES 2017–2018 guidelines specified the elevated levels as ALT > 40 U/L for men and > 31 U/L for women, AST > 37 U/L for men and > 31 U/L for women, GGT > 51 U/L for men and > 33 U/L for women, and TBIL > 1.0 mg/dL for both sexes. Detailed information is available at https://wwwn.cdc.gov/nchs/nhanes/Default.aspx.

### Exposure measurement

In each survey cycle, PFASs were measured using Online Solid Phase Extraction-High Performance Liquid ChromatographyTurbo Ion Spray-Tandem Mass Spectrometry (online SPE-HPLC-TIS-MS/MS) ^[ 3, 19] ^. Briefly, PFASs were extracted from the serum by an automated SPE procedure, separated from other components in the SPE eluate with reversed-phase HPLC, and detected by negative ion TIS-MS/MS with multiple reaction monitoring. Comprehensive details of the detection methods are available at https://wwwn.cdc.gov/nchs/nhanes/index.htm. The distribution and limits of detection (LODs) for PFASs across these cycles are illustrated in ***Supplementary Fig. 1*** (available online).

According to the NHANES analysis guideline, the serum concentration of PFAS that fell below the LOD was estimated using the LOD divided by the square root of 2 ^[ 3, 19] ^. Previous studies indicated that the bias was acceptable as long as the percentage of observation values below the LOD was not more than 30% ^[ 20– 21] ^. Therefore, our analysis was limited to the five PFASs (namely perfluorooctanoic acid [PFOA], perfluorohexane sulfonic acid [PFHxS], perfluorooctane sulfonic acid [PFOS], perfluorodecanoic acid [PFDeA], and perfluorononanoic acid [PFNA]) that had detection frequencies exceeding 70% in the samples, thus minimizing bias from these estimations. The proportions of observations at or above each LOD for PFOA, PFHxS, PFOS, PFDeA, and PFNA were 99.40%, 98.74%, 99.47%, 84.71%, and 97.76%, respectively. Note that PFDeA is often abbreviated as PFDA in some literature. For the survey cycles between 2013 and 2018, the total concentrations of PFOA and PFOS were calculated by summing the levels of their branched and linear isomers. Comprehensive information on the analytical methods and quality control/quality assurance procedures is available on the NHANES website.

### Covariates

Variables were selected as potential confounders based on the established knowledge and prior research. These included age (in years), sex (female and male), race/ethnicity background (Mexican American, Hispanic, Non-Hispanic White, Non-Hispanic Black, and Other), education level (under high school, high school or equivalent, and above high school), marital status (married, widowed, divorced, separated, never married, and cohabiting), physical activity in leisure time (min/month), smoking status (never, former, and current), diabetes mellitus (yes and no), hypertension (yes and no), and body mass index (BMI, in kg/m ^2^). Physical activity in leisure time was defined as the time spent in moderate physical activity in a typical month. Diabetes was identified using the American Diabetes Association guidelines, which include criteria such as a fasting plasma glucose level of 126 mg/dL or higher, insulin or diabetes medication use, hemoglobin A1C of 6.5% or higher, or a previous clinical diagnosis. Hypertension was determined by an average blood pressure reading of 140/90 mmHg or higher, a self-reported hypertension diagnosis, or current anti-hypertensive medication use. BMI was measured by trained examiners and calculated as weight in kilograms divided by height in meters squared.

### Statistical analysis

We calculated the mean and standard deviation, or median and inter-quartile range for continuous variables, and the number of cases ( *n*) and percentages (%) for categorical variables. For normally distributed variables, we used Student's *t*-tests to compare between groups, and for skewed variables, we used Wilcoxon rank-sum tests. For categorical variables, Chi-square tests were used to compare rates between groups. All PFASs and liver function test indicators showed right-skewed distributions, so we used natural logarithm (ln)-transformation for these variables when treating them as continuous to improve the normality of the data in statistical models. We also calculated Pearson correlation coefficients to assess the relationships among the ln-transformed concentrations of five PFASs.

To investigate the non-linear associations of PFASs with liver injury, we used the restricted cubic spline (RCS) method, adjusting for potential covariates. The Akaike information criterion (AIC) was applied to determine the most suitable knots, chosen based on the lowest AIC value ^[ 14] ^. The test for non-linearity ( *P* for non-linear) was conducted using the ANOVA function to evaluate the statistical significance of dose-dependent associations.

To analyze the association between PFAS exposure and liver injury risk, we set up three linear regression models with adjustment for different covariates. Model 1 was a crude model that had not been adjusted for covariates to investigate the extent to which the selected covariates might confuse these associations. Model 2 included adjustment for age, sex, and race/ethnicity. Model 3, building on Model 2, further adjusted for educational level, marital status, leisure-time physical activity, smoking status, diabetes mellitus, hypertension, and BMI. Additionally, we utilized multivariable logistic regression models to calculate odds ratios (ORs) and their 95% confidence intervals (CIs) to understand the association between PFAS exposure and suspected liver injury risk, considering the previously mentioned potential confounders. PFASs were treated as categorical quartiles of the increasing exposure, with the first quartile as the reference to examine any dose-response trends.

To examine the combined effects of five different PFASs on liver injury, we employed the weighted quantile sum (WQS) regression, which is suitable for handling highly correlated and collinear data. The WQS regression model is a mixed effects strategy that tests the systematic chemical exposure load based on weights determined by bootstrap sampling. The weight range is 0–1, and the sum of weights is 1. This method simulates the complexity of real-world situations more accurately. The WQS index is created based on the quartiles of chemicals and calculates the contribution of each compound to the overall effectiveness of the mixture. In short, the concentrations of all PFASs were combined into one value, assigned to a quartile, weighted through bootstrap sampling, and the final value was calculated. In the current WQS regression model, the data were randomly divided into a training set (40%) and a validation set (60%). The weights of PFASs were estimated in the training set, and the correlation between the weighted index and liver injury risk was also tested in the validation set. The WQS regression models were adjusted for variables including age, sex, race/ethnicity, education level, marital status, leisure-time physical activity, smoking status, diabetes mellitus, hypertension, and BMI. Additionally, we created positive and negative WQS indexes for all results to explain the potential different effect directions in PFASs. These two models assumed that each component of the WQS index was positively or negatively correlated with the risk of liver injury, namely positive or negative models, respectively. Finally, we performed a stratified analysis by sex (male and female), age (< 40 years and ≥ 40 years), and BMI (< 30 and ≥ 30) to further investigate the specific factors influencing the study results.

Consistent with previous recommendations, we have presented our findings without the use of sampling weights ^[ 22– 23] ^. There were two main reasons for this approach. First, adding further adjustment for variables already accounted for in the calculation of sampling weights (such as age, race/ethnicity, and sex) in our regression analyses might decrease the precision of our estimates and potentially introduce an over-adjustment bias ^[ 24] ^. Secondly, the current version of the R package for WQS regression did not support the incorporation of variables related to complex survey structures ^[ 25] ^. All our statistical analyses were conducted using Stata 17.0 (StataCorp LLC, College Station, TX, USA) and R 4.2.2 (R Foundation for Statistical Computing, Vienna, Austria). We considered a two-sided *P*-value of less than 0.05 to be statistically significant.

## Results

### Characteristics of the study participants

***Table 1*** outlines basic characteristics of the participants in the current study, stratified by sex. The study included 3520 male and 3964 female participants, with an average age of 49.9 years. Among these, men demonstrated a greater tendency towards current smoking and regular exercise, had a lower likelihood of obesity compared with women, and exhibited higher levels of both liver injury indicators and PFASs.

**Table 1:** Characteristics of the study participants and the distribution of PFASs by sex

Characteristics	Total population ( *n*=7484)	Male ( *n*=3520)	Female ( *n*=3964)	*P*
Age (years, mean±SD)	49.89±17.75	49.95±17.96	49.83±17.56	0.764
Race/ethnicity [ *n* (%)]				0.050
Mexican American	1112 (14.86)	512 (14.55)	600 (15.14)	
Hispanic	809 (10.81)	345 (9.80)	464 (11.70)	
Non-Hispanic White	2963 (39.59)	1436 (40.80)	1527 (38.52)	
Non-Hispanic Black	1554 (20.76)	726 (20.62)	828 (20.89)	
Other	1046 (13.98)	501 (14.23)	545 (13.75)	
Education [ *n* (%)]				0.160
Under high school	1751 (23.40)	855 (24.29)	896 (22.60)	
High school or equivalent	1585 (21.18)	751 (21.34)	834 (21.04)	
Above high school	4148 (55.42)	1914 (54.37)	2234 (56.36)	
Marital status [ *n* (%)]				<0.001
Married	3960 (52.91)	2084 (59.21)	1876 (47.33)	
Widowed	576 (7.70)	129 (3.67)	447 (11.28)	
Divorced	771 (10.30)	281 (7.98)	490 (12.36)	
Separated	239 (3.19)	68 (1.93)	171 (4.31)	
Never married	1377 (18.40)	696 (19.77)	681 (17.18)	
Cohabiting	561 (7.50)	262 (7.44)	299 (7.54)	
Smoking status [ *n* (%)]				<0.001
Never	4438 (59.30)	1784 (50.68)	2654 (66.95)	
Ever	1748 (23.36)	1056 (30.00)	692 (17.46)	
Current	1298 (17.34)	680 (19.32)	618 (15.59)	
BMI (kg/m ^2^, mean±SD)	29.43±7.15	28.99±6.21	29.82±7.86	<0.001
Diabetes [ *n* (%)]				0.079
Yes	4582 (61.22)	2192 (62.27)	2390 (60.29)	
No	2902 (38.78)	1328 (37.73)	1574 (39.71)	
Hypertension [ *n* (%)]				0.123
Yes	3094 (41.34)	1488 (42.27)	1606 (40.51)	
No	4390 (58.66)	2032 (57.73)	2358 (59.49)	
Physical activity in leisure time [min/month, median (IQR)]	0 (0, 642.86)	128.57 (0, 771.43)	0 (0, 514.29)	<0.001
ALT [U/L, median (IQR)]	20.00 (15.00, 27.00)	23.00 (18.00, 31.00)	18.00 (14.00, 23.00)	<0.001
AST [U/L, median (IQR)]	22.00 (19.00, 27.00)	24.00 (20.00, 28.00)	21.00 (18.00, 25.00)	<0.001
GGT [U/L, median (IQR)]	19.00 (14.00, 29.00)	23.00 (16.00, 33.00)	17.00 (12.00, 25.00)	<0.001
TBIL [mg/dL, median (IQR)]	0.60 (0.40, 0.80)	0.70 (0.50, 0.83)	0.50 (0.40, 0.70)	<0.001
FIB-4 index [median (IQR)]	1.01 (0.66, 1.50)	1.06 (0.71, 1.59)	0.95 (0.63, 1.43)	<0.001
PFOA [ng/mL, median (IQR)]	2.00 (1.27, 3.08)	2.29 (1.51, 3.40)	1.70 (1.07, 2.77)	<0.001
PFOS [ng/mL, median (IQR)]	6.50 (3.59, 11.30)	8.40 (5.10, 13.40)	5.00 (2.79, 9.30)	<0.001
PFHxS [ng/mL, median (IQR)]	1.30 (0.70, 2.30)	1.80 (1.10, 2.70)	0.99 (0.50, 1.70)	<0.001
PFDeA [ng/mL, median (IQR)]	0.20 (0.10, 0.32)	0.20 (0.11, 0.35)	0.20 (0.10, 0.30)	<0.001
PFNA [ng/mL, median (IQR)]	0.80 (0.50, 1.24)	0.85 (0.50, 1.31)	0.72 (0.40, 1.20)	<0.001
Continuous variables were presented as mean ± standard deviation (SD) or median and inter-quartile range (IQR); categorical variables were presented as *n* (%). For normally distributed variables, the Student's t-tests were used, while for skewed variables, the Wilcoxon rank-sum tests were used to compare between groups. For categorical variables, the Chi-square tests were used to compare rates between groups. Abbreviations: BMI, body mass index; ALT, alanine aminotransferase; AST, aspartate aminotransferase; GGT, gamma-glutamyltransferase; TBIL, total bilirubin; FIB-4 index, fibrosis-4 index; PFOA, perfluorooctanoic acid; PFOS, perfluorooctane sulfonic acid; PFHxS, perfluorohexane sulfonic acid; PFDeA, perfluorodecanoic acid; PFNA, perfluorononanoic acid.				

**Figure 2:**
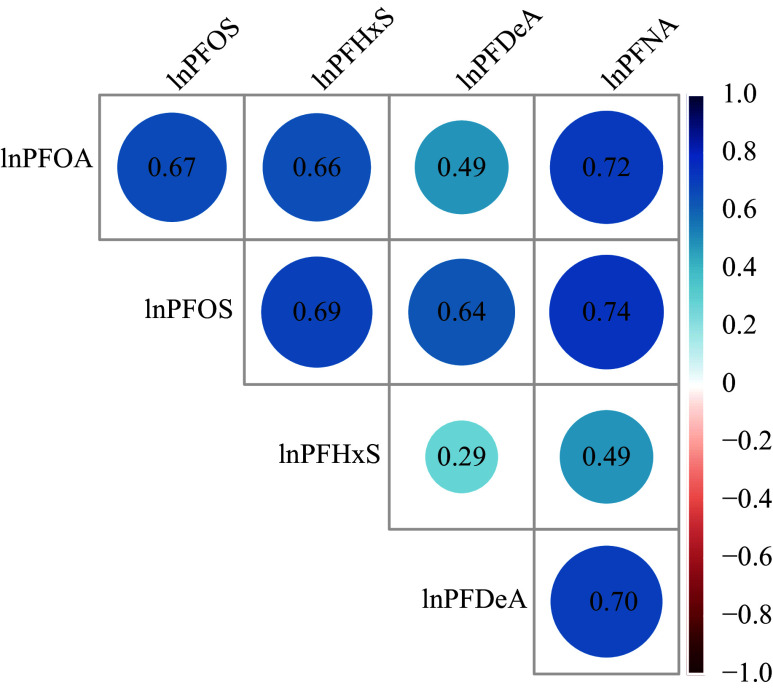
The Pearson correlation analysis on the serum concentrations of the five PFASs in the study participants. Concentrations of the PFASs were natural logarithm (ln)-transformed. Abbreviations: PFASs, per- and polyfluoroalkyl substances; PFOA, perfluorooctanoic acid; PFOS, perfluorooctane sulfonic acid; PFHxS, perfluorohexane sulfonic acid; PFDeA, perfluorodecanoic acid; PFNA, perfluorononanoic acid.

The serum detection rates for all PFASs exceeded 70%, with PFOS showing the highest median concentration at 6.50 ng/mL, followed by PFOA at 2.00 ng/mL, PFHxS at 1.30 ng/mL, PFNA at 0.80 ng/mL, and PFDeA at 0.20 ng/mL ( ***Supplementary Fig. 1***). Notably, the concentrations of all PFASs were higher in men than in women. The Pearson correlation coefficients indicated moderate to high correlation among the five PFASs, with values ranging from 0.29 to 0.74. The strongest correlation was between PFNA and PFOS, while the weakest was between PFHxS and PFDeA ( ***Fig. 2***).

### Associations between individual PFAS exposure and liver injury risk

**Figure 3:**
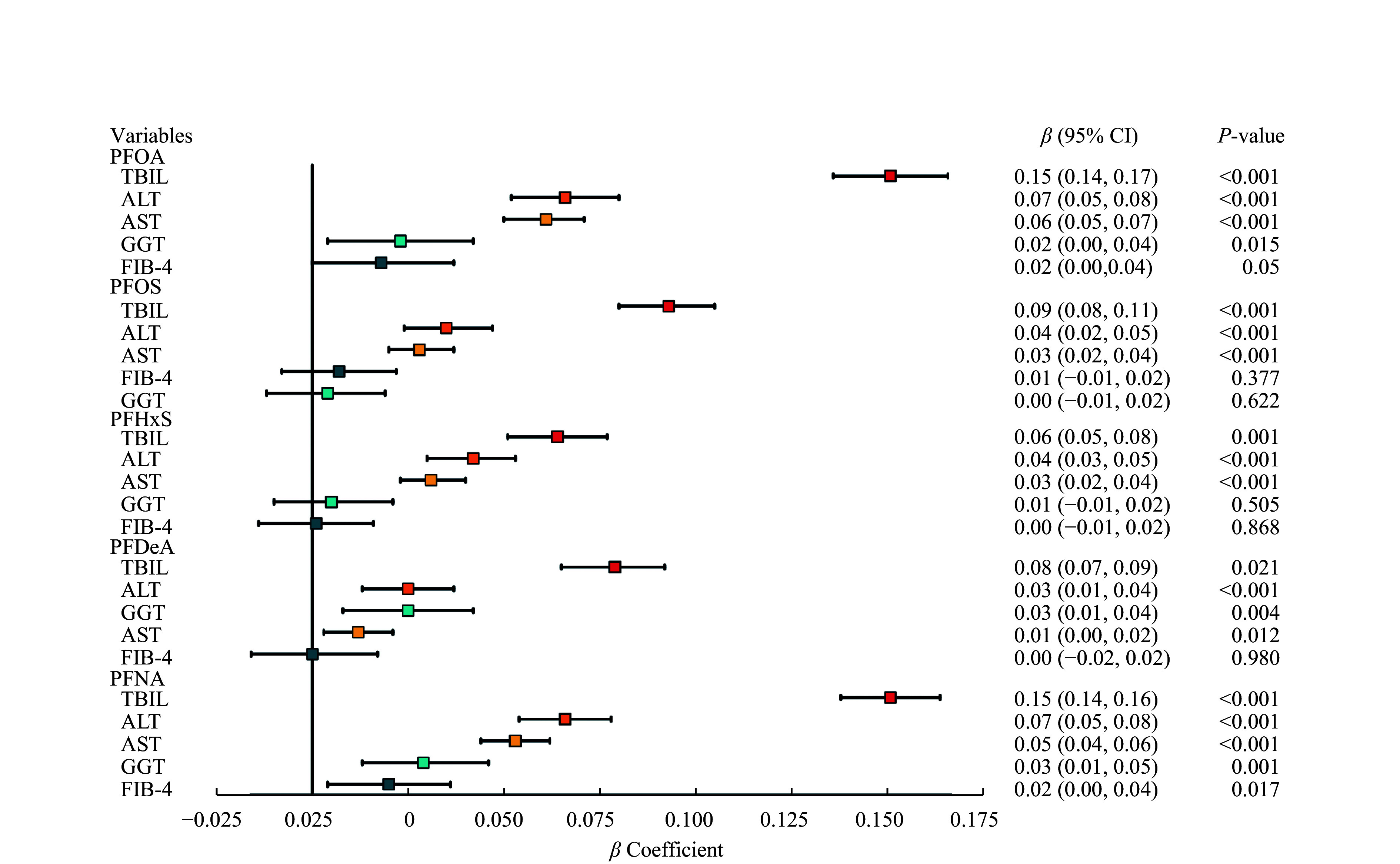
Multivariable linear regression analyses of the serum PFAS levels with liver function tests and FIB-4 index in the study participants. All models were adjusted for age, sex, race/ethnicity, education level, marital status, physical activity in leisure time, smoking status, diabetes mellitus, hypertension, and body mass index. Abbreviations: PFAS, per- and polyfluoroalkyl substance; PFOA, perfluorooctanoic acid; PFOS, perfluorooctane sulfonic acid; PFHxS, perfluorohexane sulfonic acid; PFDeA, perfluorodecanoic acid; PFNA, perfluorononanoic acid; ALT, alanine aminotransferase; AST, aspartate aminotransferase; GGT, gamma-glutamyltransferase; TBIL, total bilirubin; FIB-4 index, fibrosis-4.

We further assessed the association between PFAS exposure and liver injury risk using multiple linear regression model, as detailed in ***Fig. 3*** and ***Supplementary Table 1*** (available online). We found that the serum PFAS levels generally showed a positive association with liver injury markers, such as TBIL, ALT, and AST, in multivariable linear regression analyses. Among the five PFASs analyzed, PFOA and PFNA levels had the most significant effects on the concentrations of the aforementioned three liver enzymes. Furthermore, we observed that PFOA, PFDeA, and PFNA levels were associated with the elevated GGT levels. Only PFNA was positively associated with the FIB-4 index, while other PFASs did not show a significant association with the FIB-4 index.

In ***Supplementary Table 1***, it is noteworthy that in Model 1 without covariate adjustment, the serum PFDeA levels were not significantly correlated with the ALT levels. However, in Model 2 with adjustment for age, sex, and race/ethnicity, and further in Model 3 with adjustment for education level, marital status, physical activity in leisure time, smoking status, diabetes mellitus, hypertension, and BMI, a significant positive correlation was observed between the serum PFDeA levels and ALT levels. The positive correlation of the serum PFOS and PFHxS levels with the GGT levels was significant in Model 1, but not in Models 2 and 3. Similarly, the serum PFOS levels were significantly correlated with the FIB-4 index in Models 1 and 2, but not in Model 3. The positive correlation of the serum PFHxS and PFDeA levels with the FIB-4 index was significant in Model 1, but not in Models 2 and 3.

**Figure 4:**
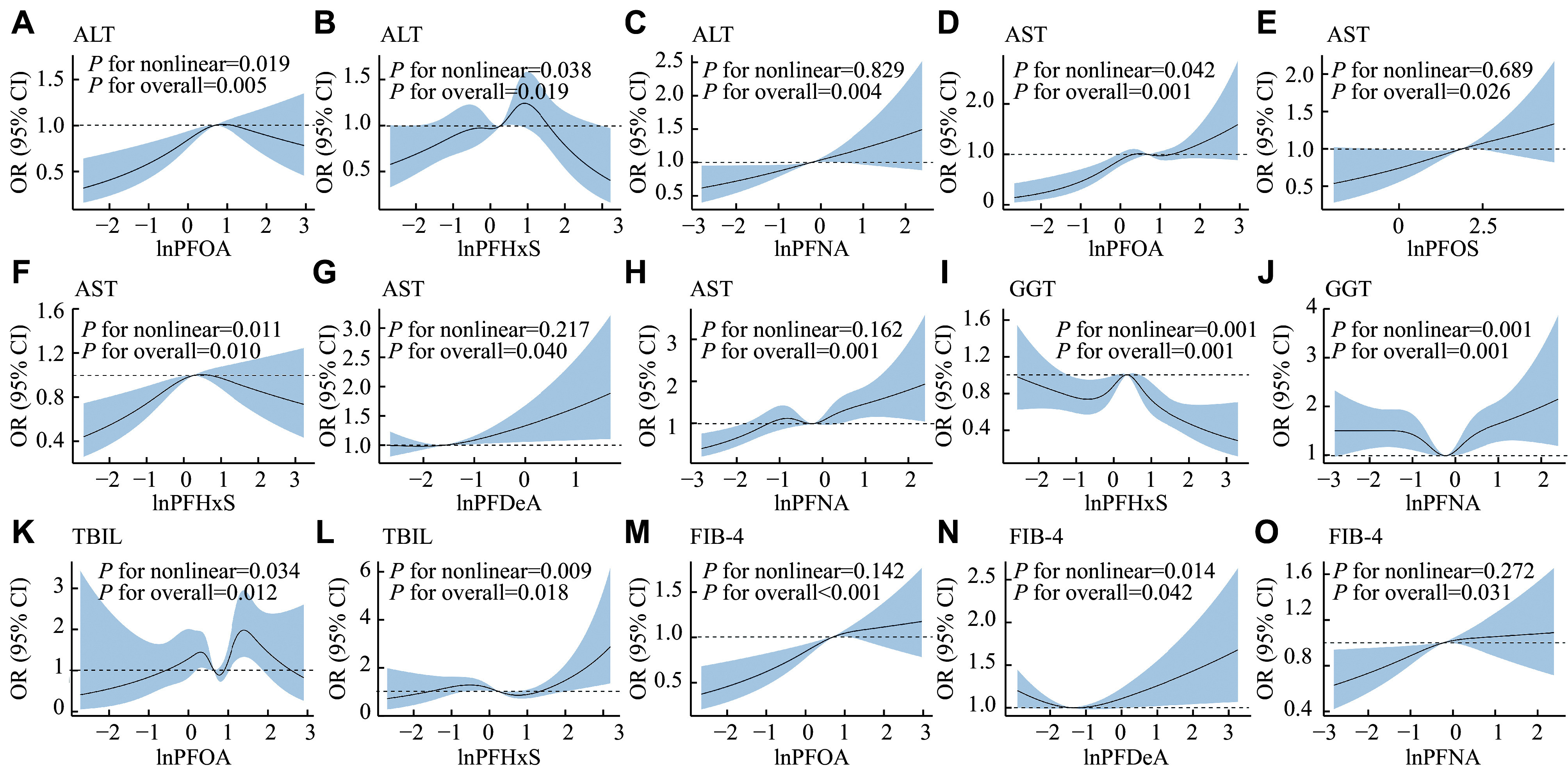
Restricted cubic spline regression analysis of the associations between the indicators of liver injury and the serum PFAS levels in the study participants. The associations of levels between ALT and PFOA (A), between ALT and PFHxS (B), between ALT and PFNA (C), between AST and PFOA (D), between AST and PFOS (E), between AST and PFHxS (F), between AST and PFDeA (G), between AST and PFNA (H), between GGT and PFHxS (I), between GGT and PFNA (J), between TBIL and PFOA (K), between TBIL and PFHxS (L), between FIB-4 index and PFOA (M), between FIB-4 index and PFDeA (N), and between FIB-4 index and PFNA (O). Concentrations of the PFASs were natural logarithm (ln)-transformed. The solid black lines represent odds ratios, and the grey-blue shaded regions represent 95% confidence intervals. The horizontal dashed line represents the reference odds ratio of 1.0. All models were adjusted for age, sex, race/ethnicity, education level, marital status, physical activity in leisure time, smoking status, diabetes mellitus, hypertension, and body mass index. Abbreviations: PFAS, per- and polyfluoroalkyl substance; PFOA, perfluorooctanoic acid; PFOS, perfluorooctane sulfonic acid; PFHxS, perfluorohexane sulfonic acid; PFDeA, perfluorodecanoic acid; PFNA, perfluorononanoic acid; ALT, alanine aminotransferase; AST, aspartate aminotransferase; GGT, gamma-glutamyltransferase; TBIL, total bilirubin; FIB-4 index, fibrosis-4 index.

***Fig. 4*** and ***Supplementary Fig. 2*** (available online) show the results of the RCS regression analysis. The U-shaped nonlinear correlations were detected between the exposure to PFNA and the level of GGT ( *P* for nonlinearity = 0.001), as well as between the exposure to PFDeA and the FIB-4 index ( *P* for nonlinearity = 0.014). The inverted U-shaped correlations of ALT, AST, and GGT levels with PFHxS levels were observed ( *P* for nonlinearity = 0.038, 0.011, and 0.001, respectively). Additionally, the PFOA levels also showed an explicitly inverted U-shaped correlation with the ALT levels ( *P* for nonlinearity = 0.019). Furthermore, positive dose-response correlations were found for exposure to PFOA and PFNA with the FIB-4 index ( *P* for nonlinearity = 0.142 and 0.272, respectively), as well as for PFOS, PFDeA, and PFNA levels with AST levels ( *P* for nonlinearity = 0.689, 0.217, and 0.162, respectively).

When dividing the PFASs into four quartiles ( ***Supplementary Fig. 3***, available online), we observed a gradual increase in the risk of severe liver fibrosis corresponding with the increasing levels of PFOA (third *vs.* first quartile: OR = 1.35, 95% CI: 1.11–1.65, *P* = 0.003; fourth *vs.* first quartile: OR = 1.32, 95% CI: 1.08–1.60, *P* = 0.007), PFOS (fourth *vs.* first quartile: OR = 1.26, 95% CI: 1.02–1.56, *P* = 0.036), and PFNA (fourth *vs.* first quartile: OR = 1.22, 95% CI: 1.01–1.48, *P* = 0.037). Compared with the lowest quartile, we also found that the fourth quartile of PFOS (OR = 1.45, 95% CI: 1.13–1.88, *P* = 0.004) and PFNA (OR = 1.31, 95% CI: 1.06–1.63, *P* = 0.014) exposure was positively associated with AST levels, the fourth quartile of PFNA (OR = 1.31, 95% CI: 1.05–1.63, *P* = 0.018) exposure was positively associated with ALT levels, and the fourth quartile of PFOA (OR = 1.56, 95% CI: 1.05–2.32, *P* = 0.027) exposure was positively associated with TBIL levels.

### Associations of the combined PFAS exposure with liver injury risk

**Figure 5:**
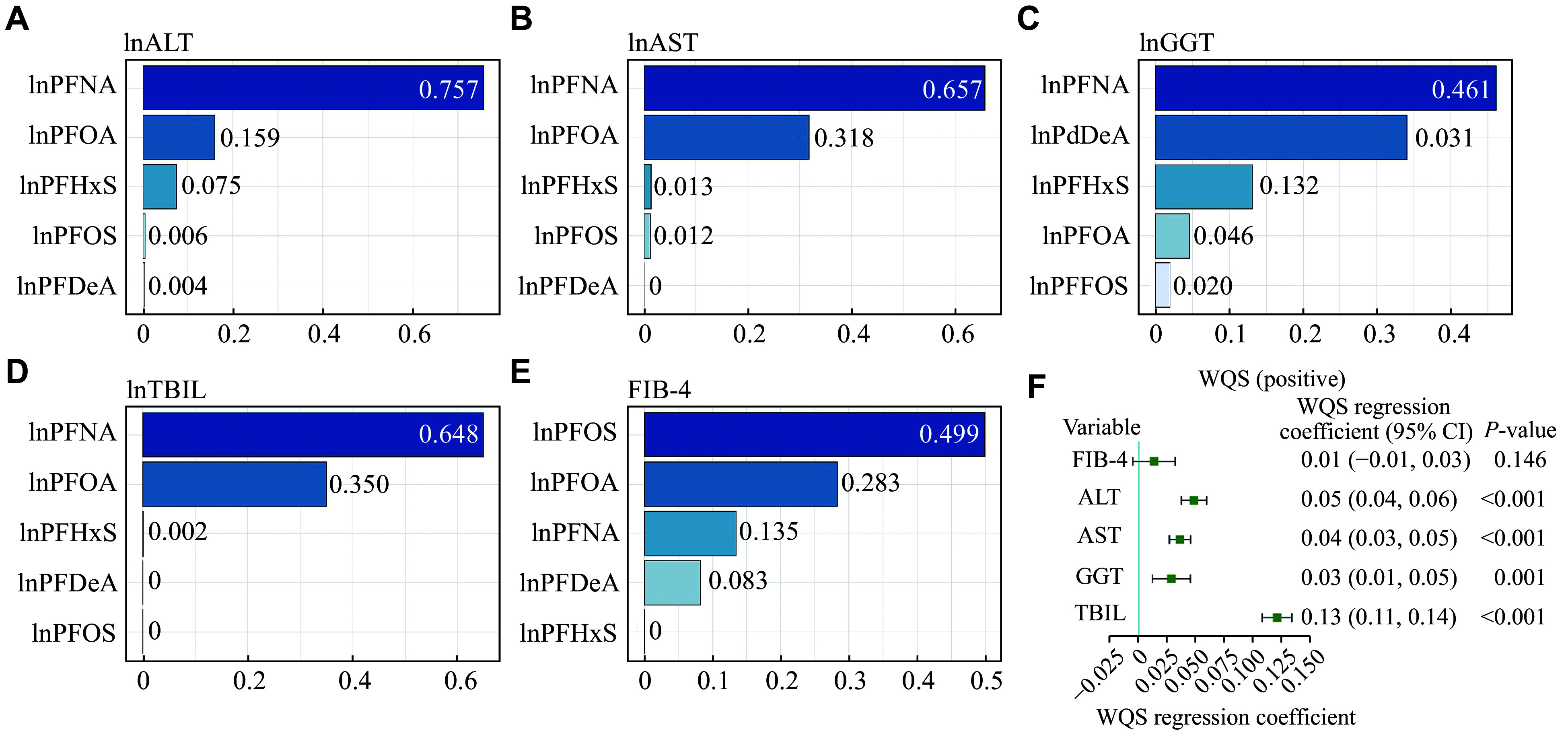
The estimated risk and weighted values of PFASs for liver injury by the WQS models. A–E: The weighted values of PFASs for ALT level (A), AST level (B), GGT level (C), TBIL level (D), and FIB-4 index (E) by the WQS models. Concentrations of the PFASs, ALT, AST, GGT, and TBIL were natural logarithm (ln)-transformed. F: The estimated risk of PFASs for liver injury by the WQS model. All models were adjusted for age, sex, race/ethnicity, education level, marital status, physical activity in leisure time, smoking status, diabetes mellitus, hypertension, and body mass index. Abbreviations: WQS, weighted quantile sum; PFASs, per- and polyfluoroalkyl substances; PFOA, perfluorooctanoic acid; PFOS, perfluorooctane sulfonic acid; PFHxS, perfluorohexane sulfonic acid; PFDeA, perfluorodecanoic acid; PFNA, perfluorononanoic acid; ALT, alanine aminotransferase; AST, aspartate aminotransferase; GGT, gamma-glutamyltransferase; TBIL, total bilirubin; FIB-4 index, fibrosis-4.

In the current study, we also investigated the collective effects of various PFAS exposures on liver injury markers through the WQS model. The results ( ***Fig. 5*** and ***Supplementary Table 2*** [available online]) showed that the WQS indexes had a significant positive correlation with liver enzyme levels, such as ALT, AST, GGT, and TBIL ( *P* < 0.001). However, the correlation with the FIB-4 index was not statistically significant ( *P* = 0.146). Notably, in the instances of positive association with ALT, AST, and TBIL, PFNA exposure had the most substantial influence on the WQS index (0.757 for ALT, 0.657 for AST, and 0.648 for TBIL, respectively), followed by the exposure to PFOA, PFHxS, PFOS, and PFDeA. In the case of GGT, PFNA exposure again showed the highest contribution to the WQS index (0.461), with the exposure to PFDeA, PFHxS, PFOA, and PFOS following in the contribution. These results were consistent with those observed in individual associations, where PFNA exposure had the most significant effect on the concentrations of liver enzymes.

### Stratified analysis

In the analyses stratified by sex ( ***Supplementary Table 3***, available online), significant associations were found between PFDeA exposure and GGT levels ( *β* = 0.03, 95% CI: 0.01–0.05, *P* = 0.007), as well as between PFNA exposure and GGT levels ( *β* = 0.03, 95% CI: 0.01–0.06, *P* = 0.003), both exclusively in females, but not in males. Age-specific analysis revealed that among participants over 40 years old, the exposure to PFOS ( *β* = 0.08, 95% CI: 0.06–0.10, *P* < 0.001), PFHxS ( *β* = 0.08, 95% CI: 0.05–0.10, *P* < 0.001), and PFDeA ( *β* = 0.03, 95% CI: 0.00–0.05, *P* = 0.032) was associated with a higher risk of liver fibrosis. This association was not observed in participants younger than 40 years. Furthermore, when analyzing individual PFASs and stratifying by BMI, notable associations between PFASs and liver injury markers were observed in obese participants, with these substances showing stronger associations with liver injury risk in obese individuals, compared with those who were not obese.

## Discussion

To our knowledge, the current study is one of the few to concurrently examine the effects of multiple PFASs on liver fibrosis levels in the general population. It is also one of the limited studies assessing the association between exposure to PFAS mixtures and liver injury-related outcomes in the U.S. population. Our findings indicated that higher serum concentrations of PFASs were associated with elevated levels of liver function biomarkers (ALT, AST, GGT, and TBIL), as well as an increased risk of liver fibrosis.

In the current study, the median concentrations of PFASs in the population were found to be lower than those reported in previous studies ^[ 11, 26] ^. One possible explanation may be that concerns about PFASs were raised earlier in the U.S. Following the voluntary phase-out of major manufacturers in the early 2000s, PFAS levels in the bloodstream of Americans decreased ^[ 27] ^. Additionally, differences in PFAS concentrations may be influenced by race-related factors.

Our findings align with most prior research showing positive associations between PFAS exposure, especially PFOA, PFOS, and PFNA, and liver injury risk. A cross-sectional study of 1404 Korean adults in the Korean National Environmental Health Survey (KoNEHS) reported positive associations of the exposure to PFOS, PFOA, PFHxS, PFNA, and PFDeA, with liver enzyme levels such as ALT, AST, and GGT ^[ 26] ^. Furthermore, a recent systematic review and meta-analysis, which included 16 cross-sectional studies, six longitudinal studies, and two mixed studies, found associations of PFOA, PFOS, and PFNA exposure with liver injury markers, based on human studies ^[ 28] ^. While the exact molecular hepatotoxicity mechanisms of PFAS are not fully understood, earlier studies showed that PFASs might cause liver toxicity in rodents ^[ 29] ^. The activation of peroxisome proliferator-activated receptor alpha (PPARα) in rodents indicated that PPARα might contribute to liver inflammation and triglyceride accumulation by activating both human and mouse PPARα and other receptors, because of their structural resemblance to fatty acids ^[ 30– 32] ^. However, in the current study population, we did not find a positive association between PFOS and PFHxS exposure and GGT levels. The evidence linking PFDeA and PFHxS exposure with GGT levels remains inconclusive and varies in both human and rodent studies, possibly due to the limited number of studies available ^[ 33] ^. More toxicological and epidemiological research is needed to better understand the potential effects of PFOS and PFHxS exposure on GGT.

A crucial discovery from the current study is the positive association between PFAS exposure and liver fibrosis. We observed significant associations of exposure to PFOA, PFOS, and PFNA with FIB-4 indexes in both linear and logistic regression models, indicating the potential hepatotoxic effects of PFASs. Furthermore, the positive correlation between PFAS exposure and the FIB-4 index indicates that prolonged exposure to PFASs may be associated with liver fibrosis, considering that these biomarkers are typically used for fibrosis assessment. Notably, considerable differences were observed across age categories, with most PFASs associated with liver fibrosis only in individuals over 40 years old. This may be because of the persistent nature of PFASs, resulting in prolonged retention and accumulation in the body. However, this association has only been reported in one cross-sectional study involving 79 children and one toxicological analysis. Therefore, future research is required to thoroughly investigate this issue ^[ 34– 35] ^.

Although the serum levels of PFNA were relatively low in the current study, they were still positively correlated with liver function biomarkers and liver fibrosis indicators, and PFNA had a higher weight in the WQS analysis. Therefore, we speculate that the general population is more sensitive to liver toxicity caused by PFNA compared with other PFASs. This finding is consistent with existing literature. Stratakis *et al* ^[ 36]^ conducted a mother-child study from the European Human Early-Life Exposome cohort and found that PFNA and PFOA exposure were the dominant contributors to the combined effects of liver injury. This indicates that the general population may be more sensitive to PFNA-induced hepatotoxicity than to other PFASs. Therefore, increasing attention and monitoring of PFNA exposure among the general population is crucial to prevent its adverse effects on liver function.

The current WQS model analysis showed that PFOS had the most substantial effect on FIB-4 indexes, a finding that diverges from the results obtained through single-PFAS-based linear regression. This discrepancy indicates that the absence of a significant association between PFOS exposure and the FIB-4 index in individual chemical assessments may be attributed to the interactions with closely related chemicals like PFHxS and PFDeA. Our findings reinforce the conclusion of a previous study, which indicated that the WQS regression model was more adept at pinpointing key factors, compared with analyses focusing on single chemicals ^[ 37] ^.

Notably, the current study highlighted a significant association between PFAS exposure and liver damage risk, with the effect being more pronounced in females. This aligns with prior findings that have indicated a higher vulnerability in females to the hepatotoxic effects of PFAS exposure ^[ 38] ^. Such differences may be attributed to variations in how each sex responds to PFAS exposure, differences in gene expression, mitochondrial functionality, the activity of microsomal enzymes, the composition of membrane lipids, and immune system reactions ^[ 39] ^. Additionally, the current study noted that while the associations between PFAS exposure and liver injury risk were observable in both obese and non-obese groups, they were significantly stronger and statistically significant only in obese individuals. Earlier research also found similar connections between exposure to PFASs and liver function markers specifically in obese subjects ^[ 40] ^. Since obesity is known to lead to fat buildup in the liver and heighten the risk of liver damage, these findings are noteworthy ^[ 41] ^. Nevertheless, the current study emphasizes the need for additional research to fully verify these results and to elucidate how PFAS exposure affects liver injury across different sexes and BMI categories.

A key advantage of the current study lies in employing three distinct models to measure and depict the individual and collective effects of PFAS exposure on liver damage. While these models themselves may not be novel, our innovation lies in the comprehensive application of these models, especially in dealing with the complexity of PFAS-exposed data. This approach enhances the reliability and solidity of the findings of the current study. Notably, the WQS, a relatively novel model for estimating the health association of mixed chemical exposures, allows for consideration of a range of highly correlated chemicals. The WQS regression is particularly effective in illustrating the individual contributions of each component in a chemical mixture, which are relatively new applications in the field of PFAS research. However, a limitation of the WQS approach is its inability to simultaneously accommodate both positive and negative effects of individual substances within a single model, because it is designed to assume a uniform direction of effect. Secondly, we not only evaluated liver injury from the perspective of liver function biomarkers but also assessed the degree of liver fibrosis. Currently, only a few studies have reported the association between exposure to PFASs and fibrosis risk, and more investigations are needed to address this issue to evaluate the potential toxic effects of PFASs on the human liver. Additionally, the current study had a relatively large sample size and was nationally representative, using the data that had been consolidated over 10 years. Finally, subgroup analyses were conducted to account for age, sex, and BMI differences in the study. The stratified analysis provides us with a more refined interpretation of the effects in specific population subsets, helping identify female, obese, and older populations who may be more susceptible to PFASs, indicating that implementing intervention measures in these populations may be more beneficial.

The current study also has some limitations. One limitation is the challenge of establishing a definitive cause-and-effect association between PFAS exposure and liver injury risk, given the cross-sectional nature of NHANES. Nevertheless, the prolonged half-lives of the PFAS compounds analyzed here make the likelihood of reverse causality less plausible. Additionally, our analysis was restricted to the five most frequently studied and persistent PFAS compounds found in populations. We did not evaluate newer, short-chain, or alternative PFAS compounds. Furthermore, although we adjusted our models for many potential confounders, there may still be residual confounders that were not taken into account. Therefore, to fully understand the associations between PFASs and liver injury, future in-depth mechanistic studies and large-scale prospective research are required.

In summary, the current study demonstrates the associations between specific PFAS exposures and the markers indicative of liver damage or liver function in American adults, with variations observed across sex, age, and BMI groups. Our findings underscore the importance of reducing occupational and everyday exposure to PFASs to mitigate their harmful health effects. The current study also adds to the expanding epidemiological evidence that indicates the potential subclinical hepatotoxicity of PFAS exposure.

## SUPPLEMENTARY DATA

Supplementary data to this article can be found online.
