# Supplementary material for: Association of exposure to per- and polyfluoroalkyl substances with liver injury in American adults
Source: J Biomed Res. 2024 May 29;38(6):628–39. doi: 10.7555/JBR.38.20240018 (PMC11629162; doi:10.7555/JBR.38.20240018)
Supplement: Supplementary file 1 — Supplementary data to this article can be found online. [file jbr-38-6-628-S1.pdf]

Available online at www.jbr-pub.org.cn

Open Access at PubMed Central

JBR

Journal of Biomedical Research, 2024 38(6): 1–4

Supplementary Data

Association of exposure to per- and polyfluoroalkyl substances with liver injury in American adults

Yuqian Yan[1,2,△], Lu Zhang[1,2,△], Xin Xu[2], Jing Lu[1,2], Xinyuan Ge[2], Maojie Liu[2], Juan Yang[1,2], Chan Tian[1,2], Zijun Ge[3], Chengxiao Yu[2], Wen Guo[1], Chunyan Ye[4,✉], Qun Zhang[1,✉]

[1]*Health Management Center, the First Affiliated Hospital of Nanjing Medical University, Nanjing, Jiangsu 210029, China;*
[2]*Department of Epidemiology, China International Cooperation Center on Environment and Human Health, Center for Global Health, School of Public Health, Nanjing Medical University, Nanjing, Jiangsu 211166, China;*
[3]*Office of Infection Management, the First Affiliated Hospital of Nanjing Medical University, Nanjing, Jiangsu 210029, China;*
[4]*Department of Liver Diseases, the Third People's Hospital of Changzhou, Changzhou, Jiangsu 213000, China.*

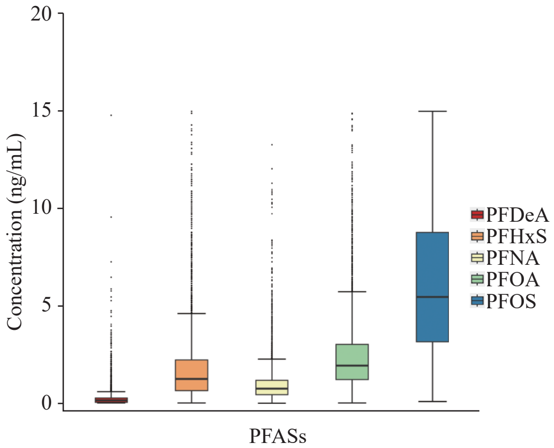

Supplementary Fig. 1 **Distribution of the serum PFAS levels in the study participants.** The LOD for each PFAS is as follows: PFOA, 0.10 ng/mL for all cycles (2009–2018); PFOS, 0.20 ng/mL for 2011–2012 and 0.10 ng/mL for 2013–2018; PFHxS, 0.10 ng/mL for all cycles; PFDeA, 0.10 ng/mL for all cycles; PFNA, 0.082 ng/mL for 2009–2011, 0.08 ng/mL for 2011–2012, and 0.10 ng/mL for 2013–2018. Abbreviations: LOD, limit of detection; PFASs, per- and polyfluoroalkyl substances; PFOA, perfluorooctanoic acid; PFOS, perfluorooctane sulfonic acid; PFHxS, perfluorohexane sulfonic acid; PFDeA, perfluorodecanoic acid; PFNA, perfluorononanoic acid.

△These authors contributed equally to this work.

✉Corresponding authors: Chunyan Ye, Department of Liver Diseases, the Third People's Hospital of Changzhou, 300 Lanling North Road, Changzhou, Jiangsu 213000, China. E-mail: 331608712@qq.com; Qun Zhang, Health Management Center, the First Affiliated Hospital of Nanjing Medical University, 300 Guangzhou Road, Nanjing, Jiangsu 210029, China. E-mail: Lucyqzhang@njmu.edu.cn.

Received: 24 January 2024; Revised: 25 March 2024; Accepted: 30 April 2024; Published online: 29 May 2024

CLC number: R114, Document code: A

The authors reported no conflict of interests.

This is an open access article under the Creative Commons Attribution (CC BY 4.0) license, which permits others to distribute, remix, adapt and build upon this work, for commercial use, provided the original work is properly cited.

© 2024 by Journal of Biomedical Research.

https://doi.org/10.7555/JBR.38.20240018

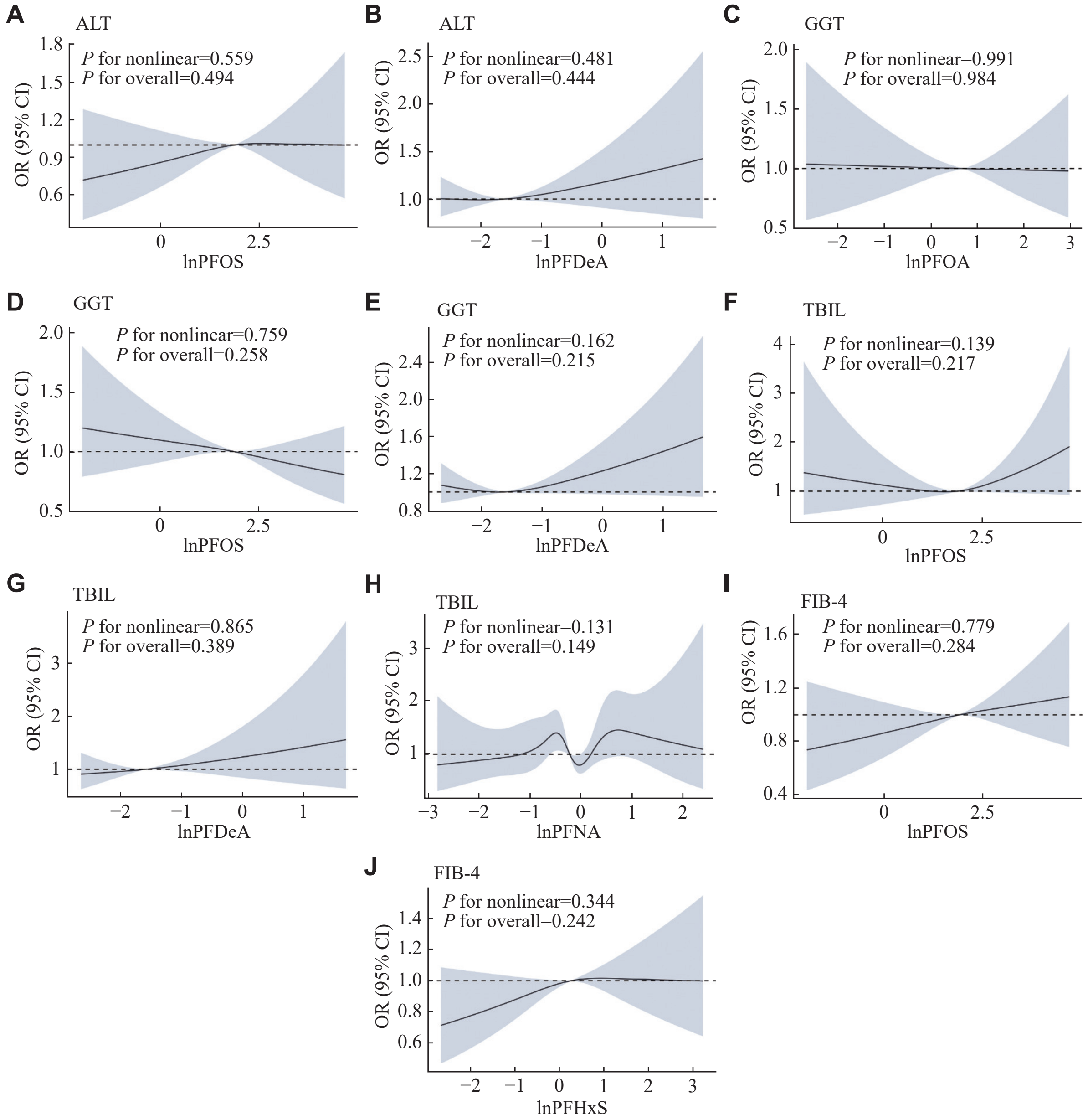

Supplementary Fig. 2 **Restricted cubic spline regression analysis of the associations between the indicators of liver injury and the serum PFAS levels in the study participants.** The associations of levels between ALT and PFOS (A), between ALT and PFDeA (B), between GGT and PFOA (C), between GGT and PFOS (D), between GGT and PFDeA (E), between TBIL and PFOS (F), between TBIL and PFDeA (G), between TBIL and PFNA (H), between FIB-4 index and PFOS (I), and between FIB-4 index and PFHxS (J). The solid black lines represent odds ratios (OR), and grey-blue shaded regions represent 95% confidence intervals (CI). The horizontal dashed line represents the reference odds ratio of 1.0. Concentrations of the PFASs were natural logarithm (ln)-transformed. All models were adjusted for age, sex, race/ethnicity, education level, marital status, physical activity in leisure time, smoking status, diabetes mellitus, hypertension, and BMI. Abbreviations: PFAS, per- and polyfluoroalkyl substance; PFOA, perfluorooctanoic acid; PFOS, perfluorooctane sulfonic acid; PFHxS, perfluorohexane sulfonic acid; PFDeA, perfluorodecanoic acid; PFNA, perfluorononanoic acid; ALT, alanine aminotransferase; AST, aspartate aminotransferase; GGT, gamma-glutamyltransferase; TBIL, total bilirubin; FIB-4 index, fibrosis-4 index.

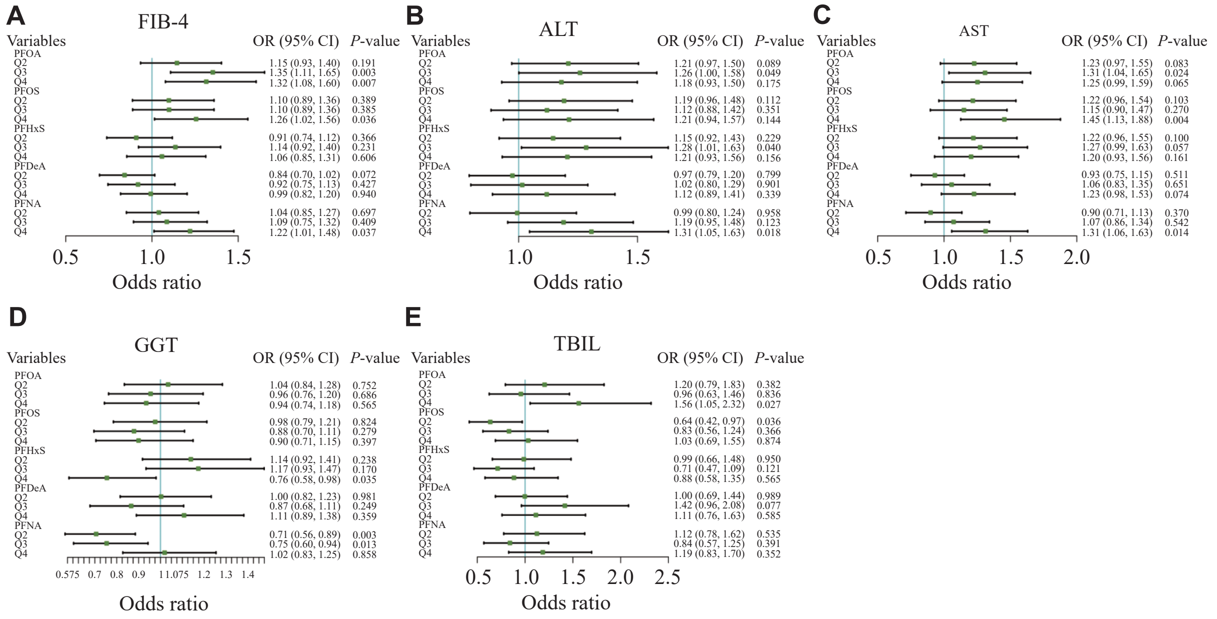

Supplementary Fig. 3 **Associations of the serum PFAS levels with liver function tests and FIB-4 index in the study participants.** Multivariable logistic regression models were used to calculate odds ratios and their 95% confidence intervals (CIs). Multivariable odds ratios for FIB-4 index (A), ALT (B), AST (C), GGT (D), and TBIL (E) according to the serum PFAS levels divided into four quartiles. All models were adjusted for age, sex, race/ethnicity, education level, marital status, physical activity in leisure time, smoking status, diabetes mellitus, hypertension, and BMI. Q2 (quartile 2), Q3 (quartile 3), and Q4 (quartile 4) represent the second, third, and fourth quartiles of exposure, respectively. Abbreviations: PFAS, per- and polyfluoroalkyl substance; PFOA, perfluorooctanoic acid; PFOS, perfluorooctane sulfonic acid; PFHxS, perfluorohexane sulfonic acid; PFDeA, perfluorodecanoic acid; PFNA, perfluorononanoic acid; ALT, alanine aminotransferase; AST, aspartate aminotransferase; GGT, gamma-glutamyltransferase; TBIL, total bilirubin; FIB-4 index, fibrosis-4; BMI, body mass index.

Supplementary Table 1 **Multivariable linear regression analyses of the serum PFAS levels with liver function tests and FIB-4 index in the study participants**

PFASs	Liver function tests	Model 1		Model 2		Model 3	
		β Coefficient (95% CI)	*P*	*β* Coefficient (95% CI)	*P*	*β* Coefficient (95% CI)	*P*
lnPFOA	lnALT	0.07 (0.05, 0.08)	<0.001	0.05 (0.04, 0.07)	<0.001	0.07 (0.05, 0.08)	<0.001
	lnAST	0.07 (0.06, 0.08)	<0.001	0.06 (0.05, 0.07)	<0.001	0.06 (0.05, 0.07)	<0.001
	lnGGT	0.04 (0.02, 0.06)	<0.001	0.01 (−0.01, 0.02)	0.624	0.02 (0.00, 0.04)	0.015
	lnTBIL	0.19 (0.17, 0.20)	<0.001	0.16 (0.15, 0.18)	<0.001	0.15 (0.14, 0.17)	<0.001
	FIB-4 index	0.19 (0.16, 0.21)	<0.001	0.03 (0.01, 0.05)	0.002	0.02 (0.00, 0.04)	0.050
lnPFOS	lnALT	0.04 (0.03, 0.05)	<0.001	0.03 (0.02, 0.04)	<0.001	0.04 (0.02, 0.05)	<0.001
	lnAST	0.04 (0.03, 0.05)	<0.001	0.03 (0.02, 0.04)	<0.001	0.03 (0.02, 0.04)	<0.001
	lnGGT	0.04 (0.03, 0.06)	<0.001	−0.01 (−0.03, 0.01)	0.155	0.00 (−0.01, 0.02)	0.622
	lnTBIL	0.12 (0.11, 0.13)	<0.001	0.10 (0.09, 0.11)	<0.001	0.09 (0.08, 0.11)	<0.001
	FIB-4 index	0.21 (0.19, 0.23)	<0.001	0.02 (0.00, 0.03)	0.036	0.01 (−0.01, 0.02)	0.377
lnPFHxS	lnALT	0.06 (0.05, 0.07)	<0.001	0.03 (0.02, 0.05)	<0.001	0.04 (0.03, 0.05)	<0.001
	lnAST	0.05 (0.04, 0.06)	<0.001	0.03 (0.02, 0.04)	<0.001	0.03 (0.02, 0.04)	<0.001
	lnGGT	0.05 (0.03, 0.06)	<0.001	−0.01 (−0.02, 0.01)	0.452	0.01 (−0.01, 0.02)	0.505
	lnTBIL	0.10 (0.09, 0.12)	<0.001	0.07 (0.06, 0.08)	<0.001	0.06 (0.05, 0.08)	<0.001
	FIB-4 index	0.17 (0.15, 0.19)	<0.001	0.01 (−0.01, 0.02)	0.246	0.00 (−0.01, 0.02)	0.868
lnPFDeA	lnALT	0.01 (−0.01, 0.02)	0.402	0.01 (0.00, 0.03)	0.034	0.03 (0.01, 0.04)	<0.001
	lnAST	0.01 (0.01, 0.02)	0.002	0.01 (0.00, 0.02)	0.011	0.01 (0.00, 0.02)	0.012
	lnGGT	0.02 (0.01, 0.04)	0.013	0.00 (−0.02, 0.02)	0.831	0.03 (0.01, 0.04)	0.004
	lnTBIL	0.09 (0.08, 0.10)	<0.001	0.09 (0.08, 0.10)	<0.001	0.08 (0.07, 0.09)	<0.001
	FIB-4 index	0.11 (0.09, 0.13)	<0.001	0.01 (0.00, 0.03)	0.139	0.00 (−0.02, 0.02)	0.980
lnPFNA	lnALT	0.06 (0.05, 0.07)	<0.001	0.06 (0.05, 0.07)	<0.001	0.07 (0.05, 0.08)	<0.001
	lnAST	0.06 (0.05, 0.07)	<0.001	0.05 (0.04, 0.06)	<0.001	0.05 (0.04, 0.06)	<0.001
	lnGGT	0.05 (0.03, 0.06)	<0.001	0.02 (0.00, 0.03)	0.049	0.03 (0.01, 0.05)	0.001
	lnTBIL	0.16 (0.15, 0.18)	<0.001	0.16 (0.14, 0.17)	<0.001	0.15 (0.14, 0.16)	<0.001
	FIB-4 index	0.16 (0.14, 0.18)	<0.001	0.03 (0.01, 0.04)	0.001	0.02 (0.00, 0.04)	0.017

Model 1 was a crude model without adjustment for covariates. Model 2 was adjusted for age, sex, and race/ethnicity. Model 3 was further adjusted for education level, marital status, physical activity in leisure time, smoking status, diabetes mellitus, hypertension, and body mass index. Concentrations of the PFASs, ALT, AST, GGT, and TBIL were natural logarithm (ln)-transformed. Abbreviations: PFAS, per- and polyfluoroalkyl substance; ALT, alanine aminotransferase; AST, aspartate aminotransferase; GGT, gamma-glutamyltransferase; TBIL, total bilirubin; FIB-4 index, fibrosis-4 index; PFOA, perfluorooctanoic acid; PFOS, perfluorooctane sulfonic acid; PFHxS, perfluorohexane sulfonic acid; PFDeA, perfluorodecanoic acid; PFNA,perfluorononanoic acid; CI, confidence interval.

Supplementary Table 2 **The associations between the WQS index and liver injury risk in the negative direction**

Outcome	WQS direction	WQS regression β coefficient	95% CI	*P*
FIB-4 index	Negative	0.015	(−0.33, 0.36)	0.178
ALT	Negative	Not estimated	–	–
AST	Negative	Not estimated	–	–
GGT	Negative	0.020	(0.00, 0.04)	0.047
TBIL	Negative	Not estimated	–	–

The model was adjusted for age, sex, race/ethnicity, education level, marital status, leisure time physical activity, smoking status, body mass index, hypertension, and diabetes. Abbreviations: ALT, alanine aminotransferase; AST, aspartate aminotransferase; GGT, gamma-glutamyltransferase; TBIL, total bilirubin; FIB-4 index, fibrosis-4 index; WQS, weighted quantile sum; CI, confidence interval.

Supplementary Table 3 **The associations between the serum PFAS levels and liver injury risk stratified by sex, age, and BMI**

PFASs	Liver function tests	Sex[a]				Age[b]				BMI[c]			
		Male (*n*=3 520)		Female (*n*=3 964)		<40 (*n*=2 447)		≥40 (*n*=5 037)		<30 (*n*=4 505)		≥30 (*n*=2 979)	
		β Coefficient (95% CI)	*P*	β Coefficient (95% CI)	*P*	β Coefficient (95% CI)	*P*	β Coefficient (95% CI)	*P*	β Coefficient (95% CI)	P	β Coefficient (95% CI)	*P*
lnPFOA	lnALT	0.05 (0.03, 0.07)	<0.001	0.05 (0.04, 0.07)	<0.001	0.06 (0.03, 0.09)	<0.001	0.05 (0.03, 0.07)	<0.001	0.05 (0.04, 0.07)	<0.001	0.08 (0.06, 0.11)	<0.001
	lnAST	0.05 (0.04, 0.07)	<0.001	0.05 (0.04, 0.07)	<0.001	0.06 (0.04, 0.07)	<0.001	0.06 (0.04, 0.07)	<0.001	0.05 (0.04, 0.06)	<0.001	0.08 (0.06, 0.10)	<0.001
	lnGGT	0.00 (−0.03, 0.03)	0.854	0.02 (0.00, 0.05)	0.084	0.03 (0.00, 0.06)	0.032	0.01 (−0.02, 0.03)	0.474	0.02 (−0.01, 0.04)	0.155	0.03 (0.00, 0.06)	0.068
	lnTBIL	0.15 (0.13, 0.18)	<0.001	0.15 (0.13, 0.17)	<0.001	0.17 (0.15, 0.20)	<0.001	0.14 (0.12, 0.16)	<0.001	0.14 (0.12, 0.16)	<0.001	0.16 (0.14, 0.19)	<0.001
	FIB-4 index	0.03 (0.00, 0.06)	0.064	0.02 (0.00, 0.04)	0.078	0.02 (0.00, 0.04)	0.034	0.08 (0.05, 0.11)	<0.001	0.01 (−0.01, 0.04)	0.363	0.04 (0.01, 0.06)	0.007
lnPFOS	lnALT	0.04 (0.02, 0.06)	<0.001	0.02 (0.00, 0.03)	0.010	0.03 (0.01, 0.06)	0.003	0.02 (0.01, 0.03)	0.006	0.03 (0.01, 0.04)	<0.001	0.05 (0.03, 0.07)	<0.001
	lnAST	0.03 (0.02, 0.05)	<0.001	0.02 (0.01, 0.03)	0.002	0.03 (0.01, 0.04)	0.001	0.02 (0.02, 0.03)	<0.001	0.02 (0.01, 0.03)	<0.001	0.04 (0.03, 0.05)	<0.001
	lnGGT	0.00 (−0.03, 0.02)	0.761	0.00 (−0.02, 0.02)	0.979	0.01 (−0.02, 0.04)	0.424	−0.01 (−0.02, 0.01)	0.582	0.01 (−0.01, 0.03)	0.497	−0.01 (−0.03, 0.02)	0.567
	lnTBIL	0.09 (0.07, 0.11)	<0.001	0.09 (0.08, 0.11)	<0.001	0.13 (0.10, 0.15)	<0.001	0.08 (0.06, 0.09)	<0.001	0.09 (0.07, 0.10)	<0.001	0.11 (0.09, 0.13)	<0.001
	FIB-4 index	0.00 (−0.02, 0.03)	0.734	0.02 (0.00, 0.03)	0.129	0.02 (0.00, 0.03)	0.065	0.08 (0.06, 0.10)	<0.001	0.01 (−0.02, 0.03)	0.602	0.02 (0.00, 0.04)	0.105
lnPFHxS	lnALT	0.03 (0.01, 0.04)	0.004	0.03 (0.01, 0.04)	<0.001	0.03 (0.01, 0.05)	0.003	0.03 (0.01, 0.04)	<0.001	0.03 (0.02, 0.05)	<0.001	0.05 (0.03, 0.07)	<0.001
	lnAST	0.02 (0.01, 0.04)	0.001	0.02 (0.01, 0.03)	<0.001	0.03 (0.01, 0.04)	0.001	0.03 (0.02, 0.04)	<0.001	0.02 (0.01, 0.03)	<0.001	0.04 (0.03, 0.06)	<0.001
	lnGGT	−0.02 (−0.05, 0.00)	0.073	0.00 (−0.02, 0.03)	0.679	0.00 (−0.02, 0.03)	0.900	0.00 (−0.02, 0.02)	0.930	0.00 (−0.02, 0.02)	0.778	0.00 (−0.02, 0.03)	0.892
	lnTBIL	0.04 (0.02, 0.06)	<0.001	0.08 (0.06, 0.10)	<0.001	0.08 (0.06, 0.10)	<0.001	0.05 (0.04, 0.07)	<0.001	0.05 (0.04, 0.07)	<0.001	0.08 (0.06, 0.10)	<0.001
	FIB-4 index	0.00 (−0.02, 0.03)	0.711	0.10 (−0.01, 0.03)	0.357	0.01 (−0.01, 0.02)	0.444	0.08 (0.05, 0.10)	<0.001	−0.01 (−0.03, 0.01)	0.309	0.02 (0.00, 0.04)	0.036
lnPFDeA	lnALT	0.03 (0.01, 0.04)	0.007	0.02 (0.00, 0.04)	0.016	0.02 (−0.01, 0.04)	0.159	0.02 (0.00, 0.03)	0.017	0.02 (0.00, 0.03)	<0.001	0.03 (0.01, 0.05)	0.004
	lnAST	0.01 (0.00, 0.03)	0.059	0.01 (0.00, 0.02)	0.175	0.00 (−0.02, 0.02)	0.904	0.01 (0.00, 0.02)	0.015	0.01 (0.00, 0.02)	0.069	0.02 (0.00, 0.04)	0.029
	lnGGT	0.01 (−0.01, 0.04)	0.353	0.03 (0.01, 0.05)	0.007	0.00 (−0.03, 0.03)	0.833	0.03 (0.01, 0.05)	0.006	0.02 (0.00, 0.04)	0.028	0.02 (−0.01, 0.05)	0.204
	lnTBIL	0.09 (0.07, 0.11)	<0.001	0.07 (0.05, 0.09)	<0.001	0.09 (0.06, 0.12)	<0.001	0.07 (0.06, 0.09)	<0.001	0.08 (0.06, 0.10)	<0.001	0.08 (0.06, 0.10)	<0.001
	FIB-4 index	0.00 (−0.03, 0.02)	0.954	0.00 (−0.02, 0.03)	0.710	0.01 (0.00, 0.03)	0.133	0.03 (0.00, 0.05)	0.032	0.01 (−0.01, 0.03)	0.527	0.00 (−0.02, 0.03)	0.921
lnPFNA	lnALT	0.06 (0.04, 0.08)	<0.001	0.06 (0.04, 0.07)	<0.001	0.06 (0.04, 0.08)	<0.001	0.05 (0.04, 0.07)	<0.001	0.06 (0.04, 0.07)	<0.001	0.08 (0.06, 0.10)	<0.001
	lnAST	0.05 (0.04, 0.07)	<0.001	0.05 (0.03, 0.06)	<0.001	0.05 (0.03, 0.06)	<0.001	0.05 (0.04, 0.06)	<0.001	0.05 (0.04, 0.06)	<0.001	0.07 (0.05, 0.08)	<0.001
	lnGGT	0.01 (−0.02, 0.03)	0.450	0.03 (0.01, 0.06)	0.003	0.03 (0.00, 0.05)	0.048	0.02 (0.00, 0.04)	0.041	0.03 (0.01, 0.05)	0.013	0.03 (0.00, 0.06)	0.031
	lnTBIL	0.15 (0.13, 0.17)	<0.001	0.15 (0.13, 0.17)	<0.001	0.18 (0.15, 0.20)	<0.001	0.14 (0.12, 0.15)	<0.001	0.15 (0.13, 0.16)	<0.001	0.16 (0.14, 0.18)	<0.001
	FIB-4 index	0.03 (0.01, 0.06)	0.013	0.02 (0.00, 0.04)	0.128	0.03 (0.01, 0.04)	0.002	0.06 (0.03, 0.09)	<0.001	0.02 (0.00, 0.04)	0.111	0.03 (0.01, 0.05)	0.012

[a]The model was adjusted for age, race/ethnicity, education level, marital status, leisure time physical activity, smoking status, body mass index, hypertension, and diabetes.
[b]The model was adjusted for sex, race/ethnicity, education level, marital status, leisure time physical activity, smoking status, body mass index, hypertension, and diabetes.
[c]The model was adjusted for age, sex, race/ethnicity, education level, marital status, leisure time physical activity, smoking status, hypertension, and diabetes.
The stratified analysis stratified by the linear regression model was performed. Concentrations of the serum PFASs, ALT, AST, GGT, and TBIL were natural logarithm (ln)-transformed. Abbreviations: PFAS, per- and polyfluoroalkyl substance; ALT, alanine aminotransferase; AST, aspartate aminotransferase; GGT, gamma-glutamyltransferase; TBIL, total bilirubin; FIB-4 index, fibrosis-4 index; PFOA, perfluorooctanoic acid; PFOS, perfluorooctane sulfonic acid; PFHxS, perfluorohexane sulfonic acid; PFDeA, perfluorodecanoic acid; PFNA, perfluorononanoic acid; BMI,body mass index; CI, confidence interval.
